# Impact of Cattaneo-Christov Heat Flux in Jeffrey Fluid Flow with Homogeneous-Heterogeneous Reactions

**DOI:** 10.1371/journal.pone.0148662

**Published:** 2016-02-09

**Authors:** Tasawar Hayat, Sumaira Qayyum, Maria Imtiaz, Ahmed Alsaedi

**Affiliations:** 1 Department of Mathematics, Quaid-I-Azam University 45320, Islamabad, 44000, Pakistan; 2 Nonlinear Analysis and Applied Mathematics (NAAM) Research Group, Department of Mathematics, Faculty of Science, King Abdulaziz University, Jeddah, 21589, Saudi Arabia; Tsinghua University, CHINA

## Abstract

Two-dimensional stretched flow of Jeffrey fluid in view of Cattaneo-Christov heat flux is addressed. Effects of homogeneous-heterogeneous reactions are also considered. Suitable transformations are used to form ordinary differential equations. Convergent series solutions are computed. Impact of significant parameters on the velocity, temperature, concentration and skin friction coefficient is addressed. Analysis of thermal relaxation is made. The obtained results show that ratio of relaxation to retardation times and Deborah number have inverse relation for velocity profile. Temperature distribution has decreasing behavior for Prandtl number and thermal relaxation time. Also concentration decreases for larger values of strength of homogeneous reaction parameter while it increases for strength of heterogeneous reaction parameter.

## Introduction

Importance of non-Newtonian fluids in boundary layer flow has increased. It is because of their extensive industrial and technological applications. The usual Navier-Stokes equation fails to describe the behavior of these kinds of flows. Mathematical formulation for such flows is in general complex. Such fluids cannot be examined by a single constitutive relationship between shear stress and rate of strain. The non-Newtonian materials are employed in applications related to biological sciences, geophysics and chemical and petroleum processes. Materials such as drilling muds, apple sauce, foams, soaps, sugar solution pastes, clay coating, ketchup, lubricant, certain oils, colloidal and suspension solutions are the non-Newtonian fluids. There are three types of non-Newtonian fluids e.g. differential, integral and rate types. Rate type fluids depicts the impact of relaxation and retardation time. Jeffrey fluid is one of the rate type materials. It shows the linear viscoelastic effect of fluid which has many applications in polymer industries. There are many examples of Jeffrey fluid including dilute polymer solution. Hayat et al. [1] analyzed the power law heat flux and heat source with Jeffrey fluid, radiation and porous medium. Hayat et al. [2] described the magnetohydrodynamic stagnation point flow of a Jeffrey nanofluid with Newtonian heating. Farooq et al. [3] examined the Newtonian heating in MHD flow of Jeffrey fluid. Hamad et al. [4] studied the thermal jump effects on boundary layer flow of a Jeffrey fluid near the stagnation point with stretching/shrinking sheet and variable thermal conductivity. Tripathi et al. [5] studied the MHD Jeffrey fluid with MHD effect on a cylindrical tube of finite length. Das [6] discussed the impact of MHD flow of Jeffrey fluid in the presence of slip and heat transfer through porous channel. Abbasi et al. [7] examined influence of heat and mass flux conditions in hydromagnetic flow of Jeffrey nanofluid. Reddy et al. [8] analyzed the flow of Jeffrey fluid between torsionally oscillating disks.

Homogeneous and heterogeneous reactions are involved in many chemically reacting systems. Some of the reactions progress slowly or absolutely not, except in the presence of catalyst. The correlation between homogeneous and heterogeneous reactions is very difficult involving the production and consumption of reactant species at different rates both within the fluid and on the catalytic surfaces. Especially the chemical reaction effect is quite significant in food processing, hydrometallurgical industry, manufacturing of ceramics and polymer production, fog formation and dispersion, chemical processing equipment design, crops damage via freezing and groves of fruit trees. Merkin [9] analyzed the viscous fluid passing through a flat plate with homogeneous-heterogeneous reactions. Chaudhry and Merkin [10] studied boundary layer flow of viscous fluid in presence of homogeneous-heterogeneous reactions. Bachok et al. [11] analyzed homogeneous-heterogeneous reactions in stagnation point flow towards a stretching sheet. Khan and Pop [12] investigated effects of homogeneous-heterogeneous reactions in the flow of viscoelastic fluid towards a stretching sheet. Kameswaran et al. [13] discussed the flow of nanofluid over a porous stretching sheet with homogeneous-heterogeneous reactions. Newtonian heating in presence of carbon nanotube and homogeneous-heterogeneous reactions are illustrated by Hayat et al. [14]. MHD flow of nanofluid with homogeneous-heterogeneous reactions and velocity slip is analyzed by Hayat et al. [15].

In industrial and engineering processes the heat transfer mechanism is very useful including nuclear reactor for cooling, energy production space cooling, biomedical applications such as heat conduction in tissues and magnetic drug targeting etc. Mechanism of heat transfer has been extensively described by classical Fourier heat conduction law [16]. However it has a major limitation that it yields a parabolic energy equation which indicates that initial disturbance is instantly experienced by the medium under consideration. This feature is referred in literature as “Paradox of heat conduction”. To overcome this situation, various researchers have proposed modifications in the Fourier's heat conduction law. Cattaneo [17] modified this law through the inclusion of relaxation time for heat flux which is defined as a time required establishing heat conduction once the temperature gradient is imposed. Equation of motion of a phonon gas and non-Fourier heat conduction has been obtained by Cao and Guo [18]. Christov [19] further modified the Cattaneo's model by replacing the ordinary derivative with the Oldroyd's upper convected derivative. Tibullo and Zampoli [20] examined the incompressible fluids reactions for Cattaneo-Christov heat conduction model. Straughan [21] applied Cattaneo-Christov thermal convection in horizontal layer of incompressible Newtonian fluid under the effect of gravity. Ciarletta and Straughan [22] studied the Cattaneo-Christov equations structural stability and uniqueness. Dong et al. [23] examined dynamical analysis of non-Fourier heat conduction and its application in nanosystems. Numerical studies on damping of thermal waves have been derived by Zhang et al. [24]. Han et al. [25] described the Cattaneo-Christov heat flux model in flow of Maxwell fluid. Mustafa [26] discussed the upper convected flow of Maxwell fluid in presence of rotation and Cattaneo Christov heat flux.

The main purpose of present paper is to investigate the steady two-dimensional flow of Jeffrey fluid over a linearly stretching sheet. Effects of Cattaneo-Christov heat flux and homogeneous-heterogeneous reaction are clearly focused. Here we develop series solutions using homotopy analysis method [27–33]. Convergent series solutions are determined. Graphs are plotted and examined for the effects of interesting parameters on the velocity, temperature, concentration and skin friction coefficient.

## Problems Formulation

We consider steady two-dimensional flow of Jeffrey fluid in the presence of Cattaneo-Christov heat flux. Fluid flow is induced by a linear stretching sheet. Sheet is at constant temperature *T*_*w*_ and temperature far away from the sheet is *T*_∞_ (i.e *T*_*w*_ ≥ *T*_∞_) Flow analysis is carried out subject to homogeneous—heterogeneous reactions. Homogeneous reaction for cubic autocatalysis can be expressed as follows:

$$A+2B\to 3B,\;\text{rate}=k_{c}ab^{2},$$ (1)

while first order isothermal reaction on the catalyst surface is presented in the form

$$A\to B,\;\text{rate}=k_{s}a.$$ (2)

Here *a* and *b* are the concentrations of the chemical species *A* and *B* and *k*_*c*_ and *k*_*s*_ are the rate constants. We assume that both reaction processes are isothermal. The conservation laws of mass, momentum, energy and concentration governing the present flow can be written below:

$$\frac{\partial u}{\partial x}+\frac{\partial v}{\partial y}=0,$$ (3)

$$u\frac{\partial u}{\partial x}+v\frac{\partial u}{\partial y}=\frac{\nu }{1+\alpha }\left(\frac{\partial^{2}u}{\partial y^{2}}+\lambda_{1}\left(u\frac{\partial^{3}u}{\partial x\partial y^{2}}+v\frac{\partial^{3}u}{\partial y^{3}}-\frac{\partial u}{\partial x}\frac{\partial^{2}u}{\partial y^{2}}+\frac{\partial u}{\partial y}\frac{\partial^{2}u}{\partial y\partial x}\right)\right),$$ (4)

$$\rho c_{p}\left(u\frac{\partial T}{\partial x}+v\frac{\partial T}{\partial y}\right)=-\nabla .q,$$ (5)

$$u\frac{\partial a}{\partial x}+v\frac{\partial a}{\partial y}=D_{A}\frac{\partial^{2}a}{\partial y^{2}}-k_{c}ab^{2},$$ (6)

$$u\frac{\partial b}{\partial x}+v\frac{\partial b}{\partial y}=D_{B}\frac{\partial^{2}b}{\partial y^{2}}+k_{c}ab^{2},$$ (7)

where (*u*, *v*) are the velocities along (*x*, *y*) directions respectively, *ν* for kinematic viscosity, *T* for temperature, *c*_*p*_ for specific heat, *ρ* for fluid density, *α* for ratio of relaxation to retardation times, *λ*_1_ for retardation time and **q** the heat flux satisfying the relation

$$q+\lambda_{2}\left(\frac{\partial q}{\partial t}+V.\nabla q-q.\nabla V+(\nabla .V)q\right)=-k\nabla T,$$ (8)

in which *λ*_2_ is the thermal relaxation time and *k* the fluid thermal conductivity. Following Christov [19], we omit **q** by using Eqs (5) and (8) and obtain

$$u\frac{\partial T}{\partial x}+v\frac{\partial T}{\partial y}=\frac{k}{\rho c_{p}}\frac{\partial^{2}T}{\partial y^{2}}-\lambda_{2}(u^{2}\frac{\partial^{2}T}{\partial x^{2}}+v^{2}\frac{\partial^{2}T}{\partial y^{2}}+2uv\frac{\partial^{2}T}{\partial x\partial y}+\left(u\frac{\partial u}{\partial x}+v\frac{\partial u}{\partial y}\right)\frac{\partial T}{\partial x}+\left(u\frac{\partial v}{\partial x}+v\frac{\partial v}{\partial y}\right)\frac{\partial T}{\partial y}).$$ (9)

The subjected boundary conditions are

$$\begin{array}{l} \;u=U_{w}=cx,\;v=0,\;T=T_{w},\;D_{A}\frac{\partial a}{\partial y}=k_{s}a,\;D_{B}\frac{\partial b}{\partial y}=-k_{s}a\;\text{at}\;y\to 0,\\ u\to 0,\;T\to T_{\infty },\;a\to a_{0},\;b\to 0\;\text{when}\;y\to \infty , \end{array}$$ (10)

where *D*_*A*_ and *D*_*B*_ are the diffusion coefficients and *a*_0_ is positive dimensional constant. Employing transformations

$$\eta =\sqrt{\frac{c}{\nu }}y,\;u=cxf^{\prime }(\eta ),\;v=-\sqrt{c\nu }f(\eta ),\;\theta =\frac{T-T_{\infty }}{T_{w}-T_{\infty }},\;a=a_{0}g(\eta ),\;b=a_{0}h(\eta ),$$ (11)

continuity equation is satisfied automatically and Eqs (4), (6), (7), (9) and (10) take the forms:

$$f^{\prime \prime \prime }+(1+\alpha )(ff^{\prime \prime }-f^{\prime^{2}})+\beta (f^{\prime \prime^{2}}-ff^{\prime \prime \prime \prime })=0,$$ (12)

$$\frac{1}{\text{Pr}}\theta^{\prime \prime }+\theta^{\prime }f-\gamma \left(f^{2}\theta^{\prime \prime }+ff^{\prime }\theta^{\prime }\right)=0,$$ (13)

$$\frac{1}{Sc}g^{\prime \prime }+fg^{\prime }-k_{1}gh^{2}=0,$$ (14)

$$\frac{\delta }{Sc}h^{\prime \prime }+fh^{\prime }+k_{1}gh^{2}=0,$$ (15)

$$f^{\prime }(0)=1,\;f^{\prime }(\infty )\to 0,\;f(0)=0,\;\theta (0)=1,\;\theta (\infty )\to 0,\;g^{\prime }(0)=k_{2}g(0),\;\delta h^{\prime }(0)=-k_{2}g(0),$$ (16)

where Pr is for Prandtl number, *β* for Deborah number, *γ* for thermal relaxation time, *Sc* for Schmidt number, *k*_1_ and *k*_2_ for measure of strength of homogeneous and heterogeneous reactions respectively and *δ* for ratio of diffusion coefficient. These parameters are defined as follows:

$$\beta =\lambda_{1}c,\;\text{Pr}=\frac{\rho c_{p}\nu }{k},\;\gamma =\lambda_{2}c,\;Sc=\frac{\nu }{D_{A}},\;k_{1}=\frac{k_{c}a_{0}^{2}}{c},\;\delta =\frac{D_{B}}{D_{A}},\;k_{2}=\frac{k}{D_{A}a_{0}}\sqrt{\frac{c}{\nu }}.$$ (17)

Here it is assumed that diffusion coefficients of chemical species *A* and *B* are of a comparable size. Through this we assume that *D*_*A*_ and *D*_*B*_ are same, i.e. *δ* = 1 and thus:

g(η)+h(η)=1. (18)

Now Eqs 14 and 15 yield

$$\frac{1}{Sc}g^{\prime \prime }+fg^{\prime }-k_{1}g{\left(1-g\right)}^{2}=0,$$ (19)

with boundary conditions

$$g^{\prime }(0)=k_{2}g(0),\;g(\infty )=1.$$ (20)

Skin friction coefficient in dimensional form is

$$C_{fx}=\frac{\tau_{w}}{\frac{\rho }{2}u_{w}^{2}(x)};\;\tau_{w}={\frac{\mu }{1+\alpha }\left[\frac{\partial u}{\partial y}+\beta \left(u\frac{\partial^{2}u}{\partial x\partial y}+u\frac{\partial^{2}v}{\partial x^{2}}+v\frac{\partial^{2}u}{\partial y^{2}}\right)\right]|}_{y=0},$$ (21)

where *τ*_*w*_ is the shear stress. Skin friction coefficient in dimensionless form along the *x* – direction is defined as follows:

$$\frac{C_{fx}{\text{Re}}_{x}^{0.5}}{2}=\frac{1}{1+\alpha }[f^{\prime \prime }(0)+\beta (f^{\prime }(0)f^{\prime \prime }(0)-f(0)f^{\prime \prime \prime }(0))].$$ (22)

Exact analytical solution of Eq (12) is [31]

$$f(\eta )=\frac{1-\text{exp}(-A\eta )}{A},$$ (23)

where

$$A=\sqrt{\frac{1+\alpha }{1+\beta }}.$$ (24)

So Eqs (13) and (19) takes the form

$$\frac{1}{\text{Pr}}\theta^{\prime \prime }+\left(\frac{1-\text{exp}(-A\eta )}{A}\right)\theta^{\prime }-\gamma \left({\left(\frac{1-\text{exp}(-A\eta )}{A}\right)}^{2}\theta^{\prime \prime }+\text{exp}(-A\eta )\left(\frac{1-\text{exp}(-A\eta )}{A}\right)\theta^{\prime }\right)=0,$$ (25)

$$\frac{1}{Sc}g^{\prime \prime }+\left(\frac{1-\text{exp}(-A\eta )}{A}\right)g^{\prime }-k_{1}g{\left(1-g\right)}^{2}=0,$$ (26)

## Homotopic Solutions

### 3.1. Zeroth-Order Deformation Equations

Since exact solution for velocity is given in Eqs (23) and (24). However the exact solutions for the temperature and concentration related systems (Eqs (25), (26) and conditions (16), (20)) are not possible. Thus homotopy analysis method is implemented to get analytical solution of considered problem. Initial guesses and auxiliary linear operators are taken as follows:

$$\theta_{0}(\eta )=\text{exp}(-\eta ),\;g_{0}(\eta )=1-\frac{1}{2}\text{exp}(-k_{2}\eta ),$$ (27)

$$L_{\theta }=\theta^{\prime \prime }-\theta ,\;L_{g}=g^{\prime \prime }-g,$$ (28)

with

Lθ[c1eη+c2e−η]=0,Lg[c3eη+c4e−η]=0, (29)

in which *c*_*i*_ (*i* = 1 − 4) are the constants.

If *q* ∈ [0,1] indicates the embedding parameter and ℏ_*θ*_ and ℏ_*g*_ are the non-zero auxiliary parameters then the zeroth order deformation problems are

$$(1-q)L_{\theta }\left[\vartheta (\eta ,q)-\theta_{0}(\eta )\right]=q\hbar_{\theta }N_{\theta }[\vartheta (\eta ,q)],$$ (30)

$$(1-q)L_{g}\left[G(\eta ,q)-g_{0}(\eta )\right]=q\hbar_{g}N_{g}[G(\eta ,q)],$$ (31)

ϑ(0,q)=1,ϑ(∞,q)=0, (32)

$$G^{\prime }(0,q)=k_{2}G(0,q),\;G^{\prime }(\infty ,q)=1,$$ (33)

where the nonlinear differential operators **N**_*g*_ and **N**_*θ*_ are given by

$$N_{\theta }\left[\vartheta (\eta ,q)\right]=\frac{1}{\text{Pr}}\frac{\partial^{2}\vartheta (\eta ,q)}{\partial \eta^{2}}+\left(\frac{1-\text{exp}(-A\eta )}{A}\right)\frac{\partial \vartheta (\eta ,q)}{\partial \eta }-\gamma \left[\begin{array}{l} {\left(\frac{1-\text{exp}(-A\eta )}{A}\right)}^{2}\frac{\partial^{2}\vartheta (\eta ,q)}{\partial \eta^{2}}\\ +\text{exp}(-A\eta )\left(\frac{1-\text{exp}(-A\eta )}{A}\right)\frac{\partial \vartheta (\eta ,q)}{\partial \eta } \end{array}\right],$$ (34)

$$N_{g}\left[G(\eta ,q)\right]=\frac{1}{Sc}\frac{\partial^{2}G(\eta ,q)}{\partial \eta^{2}}+\left(\frac{1-\text{exp}(-A\eta )}{A}\right)\frac{\partial G(\eta ,q)}{\partial \eta }-k_{1}G(\eta ,q)-k_{1}{\left(G(\eta ,q)\right)}^{3}+2k_{1}{\left(G(\eta ,q)\right)}^{2}.$$ (35)

### 3.2. m^*th*^ Order Deformation Equations

The m^*th*^ order deformation equations are

$$L_{\theta }\left[\theta_{m}(\eta )-\chi_{m}\theta_{m-1}(\eta )\right]=\hbar_{\theta }R_{\theta ,m}(\eta ),$$ (36)

$$L_{g}\left[g_{m}(\eta )-\chi_{m}g_{m-1}(\eta )\right]=\hbar_{g}R_{g,m}(\eta ),$$ (37)

$$\theta (0)=\theta (\infty )=g_{m}(\infty )=\frac{\partial g_{m}(0)}{\partial \eta }-k_{2}g_{m}(0)=0,$$ (38)

where the functions **R**_*θ*,*m*_(*η*) and **R**_*g*,*m*_(*η*) have the following forms:

$$R_{\theta ,m}(\eta )=\frac{1}{\text{Pr}}\theta_{m-1}^{\prime \prime }+\left(\frac{1-\text{exp}(-A\eta )}{A}\right)\theta_{m-1}^{\prime }-\gamma \left[{\left(\frac{1-\text{exp}(-A\eta )}{A}\right)}^{2}\theta_{m-1}^{\prime \prime }+\text{exp}(-A\eta )\left(\frac{1-\text{exp}(-A\eta )}{A}\right)\theta_{m-1}^{\prime }\right],$$ (39)

$$R_{g,m}\left(\eta \right)=\frac{1}{Sc}g_{m-1}^{\prime \prime }+\left(\frac{1-\text{exp}(-A\eta )}{A}\right)g_{m-1}^{\prime }+\sum_{k=0}^{m-1}\left(2k_{1}g_{m-1-k}g_{k}-k_{1}g_{m-k-1}\sum_{l=0}^{k}g_{k-l}g_{l}\right)-k_{1}g_{m-1},$$ (40)

$$\chi_{m}=\{\begin{array}{c} 0,\;m\leq 1\\ 1,\;m>1 \end{array}.$$ (41)

The general solutions (*θ*_*m*_, *g*_*m*_) comprising the special solutions ($\theta_{m}^{*}$, $g_{m}^{*}$) are

$$\begin{array}{c} \theta_{m}(\eta )=\theta_{m}^{*}(\eta )+c_{1}e^{\eta }+c_{2}e^{-\eta },\\ g_{m}(\eta )=g_{m}^{*}(\eta )+c_{3}e^{\eta }+c_{4}e^{-\eta }, \end{array}$$ (42)

where the constants *c*_*i*_ (*i* = 1 − 4) through the boundary conditions (38) have the values

c2=−θm*(0),c4=11+k2(∂gm*(0)∂η−k2gm*(0)),c1=c3=0. (43)

## Convergence Analysis

**Fig 1:**
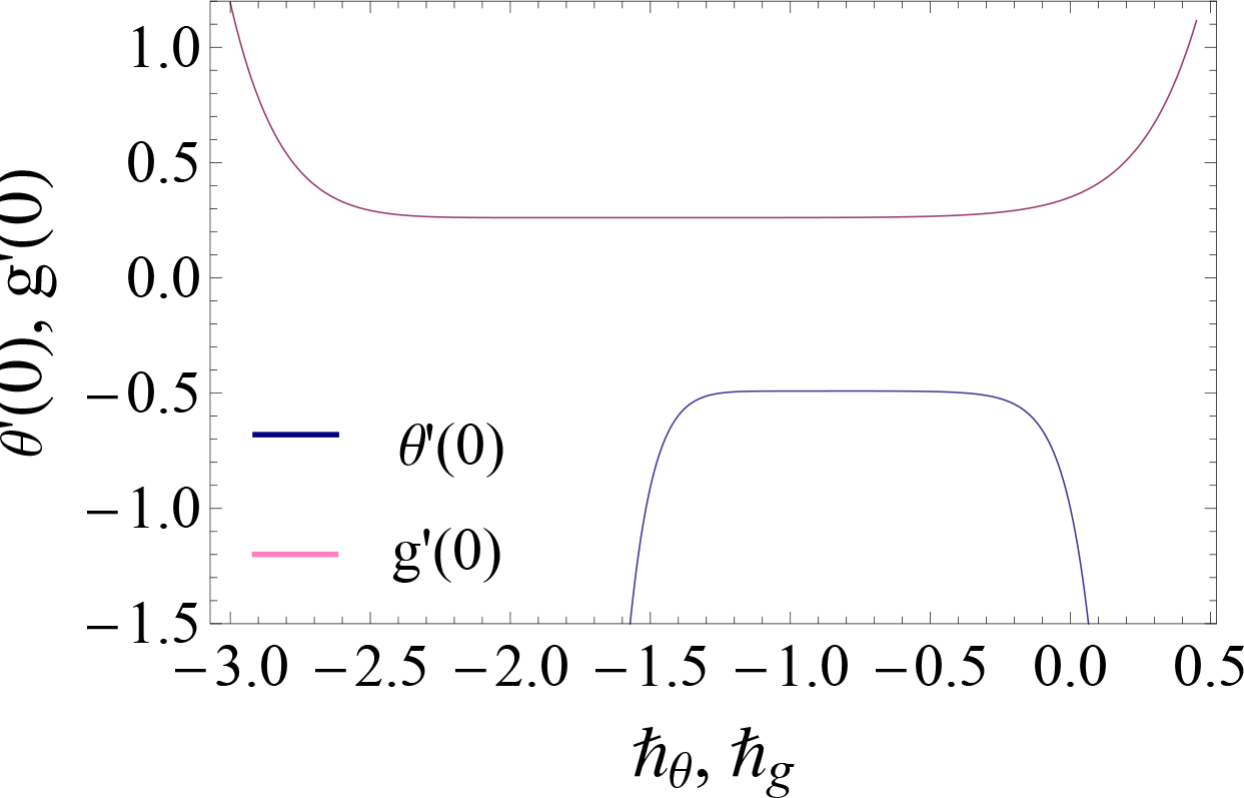
ℏ – curves for *θ*′(0) and *g*′(0) when *α* = 0.2, *β* = 0.1, Pr = *γ* = 0.7 = *k*_1_ = *k*_2_ and *Sc* = 1.

The method of homotopy analysis gives us opportunity and a simpler way to adjust and control the convergence of the series solutions. The auxiliary parameters ℏ_*θ*_ and ℏ_*g*_ have much importance for the series solution convergence. For that purpose the ℏ – curves at 10th order of approximations are plotted (see Fig 1). Admissible values of auxiliary parameters are −1.2 ≤ ℏ_*θ*_ ≤ −0.5 and −2 ≤ ℏ_*g*_ ≤ −0.5. Also the HAM solutions converge in the full range of *η* (0 ≤ *η* ≤ *∞*) where ℏ_*θ*_ = −1 and ℏ_*g*_ = −1.5.

Table 1 demonstrates the convergence of velocity, temperature and concentration equations. It is noted that 12th and 15th order of approximations are enough for the convergence of *θ*′(0) and *g*′(0).

**Table 1:** Convergence of solutions when *β* = 0.1, *α* = 0.2, *γ* = Pr = 0.7 = *k*_1_ = *k*_2_ and *Sc* = 1.

Order of approximation	−*θ*′(0)	*g*′(0)
1	0.45238	0.28048
4	0.49606	0.26213
10	0.49112	0.26099
12	0.49114	0.26103
15	0.49114	0.26104
20	0.49114	0.26104
25	0.49114	0.26104
30	0.49114	0.26104
35	0.49114	0.26104
40	0.49114	0.26104

## Results and Discussion

In this section Figs (2–14) and Table 2 show the behavior of various parameters on the velocity, temperature, concentration and skin friction coefficient.

**Fig 2:**
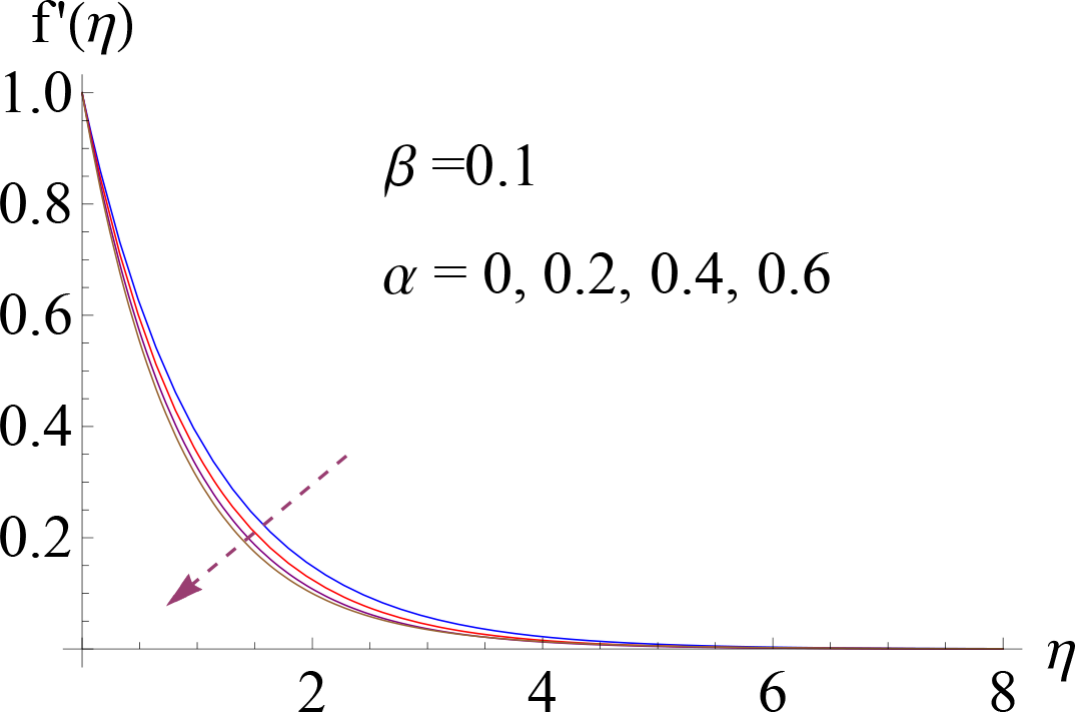
Impact of *α* on *f*′(*η*).

**Fig 3:**
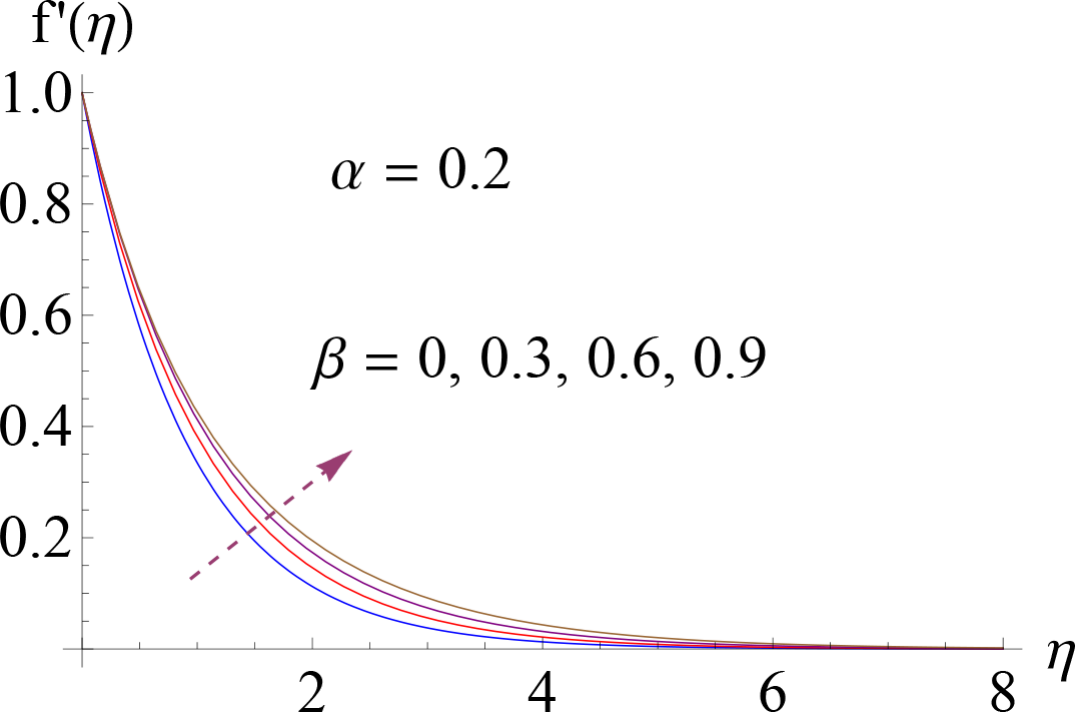
Impact of *β* on *f*′(*η*).

**Fig 4:**
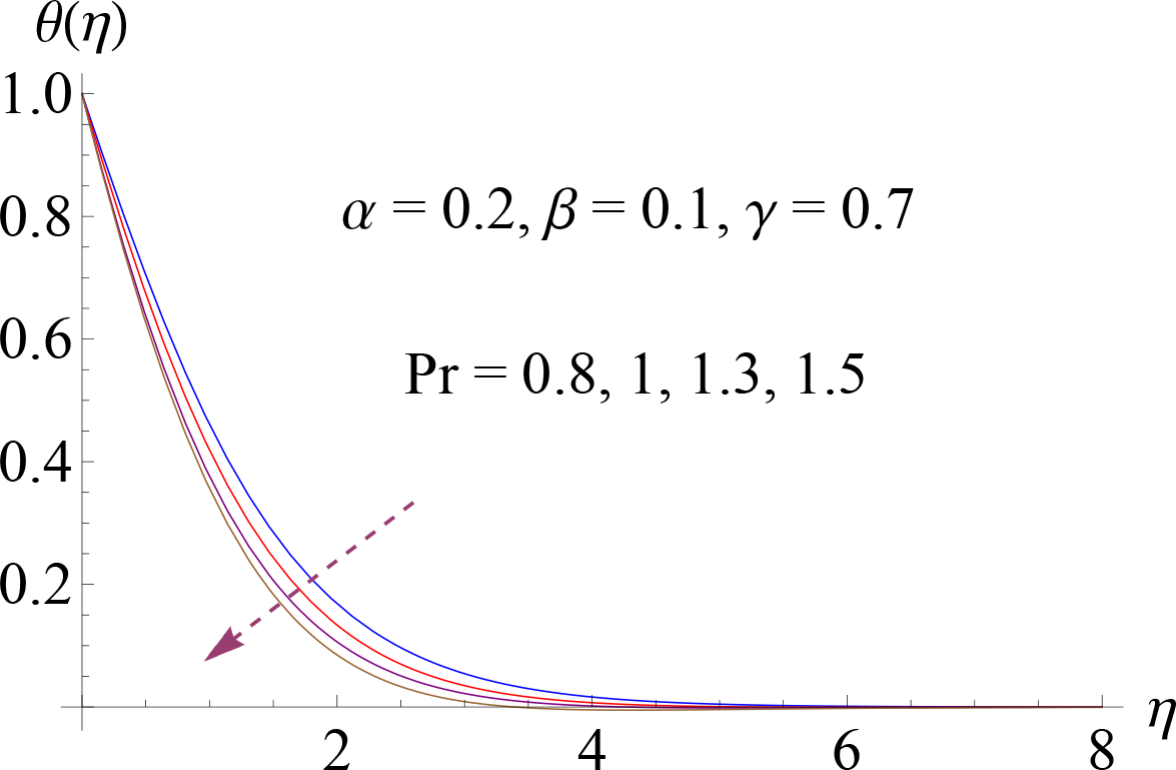
Impact of Pr on *θ*(*η*).

**Fig 5:**
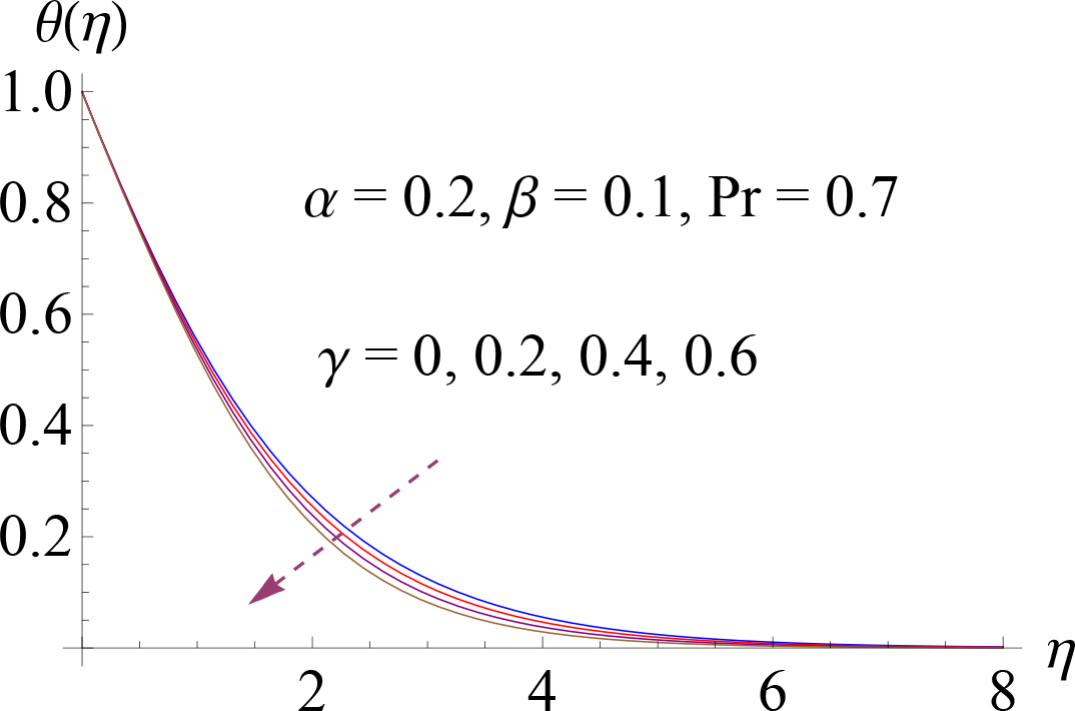
Impact of *γ* on *θ*(*η*).

**Fig 6:**
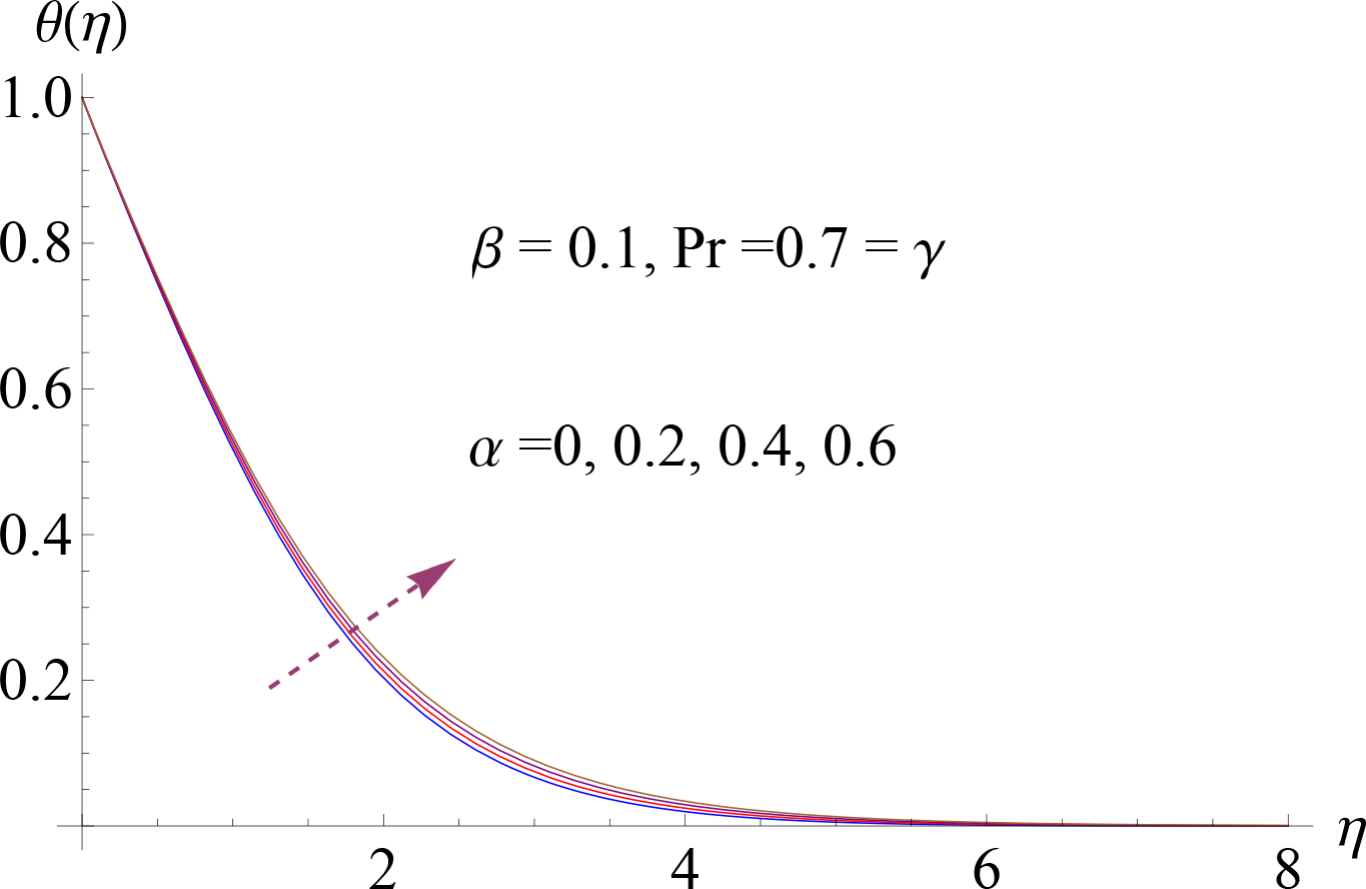
Impact of *α* on *θ*(*η*).

**Fig 7:**
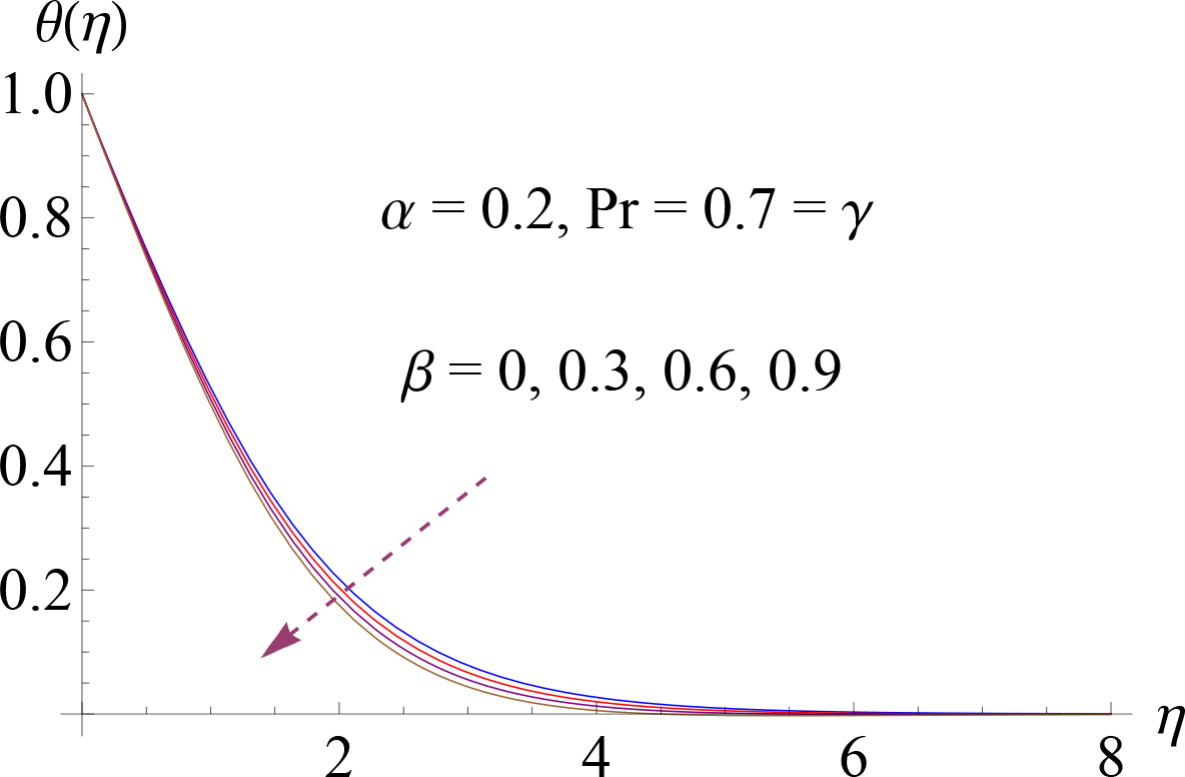
Impact of *β* on *θ*(*η*).

**Fig 8:**
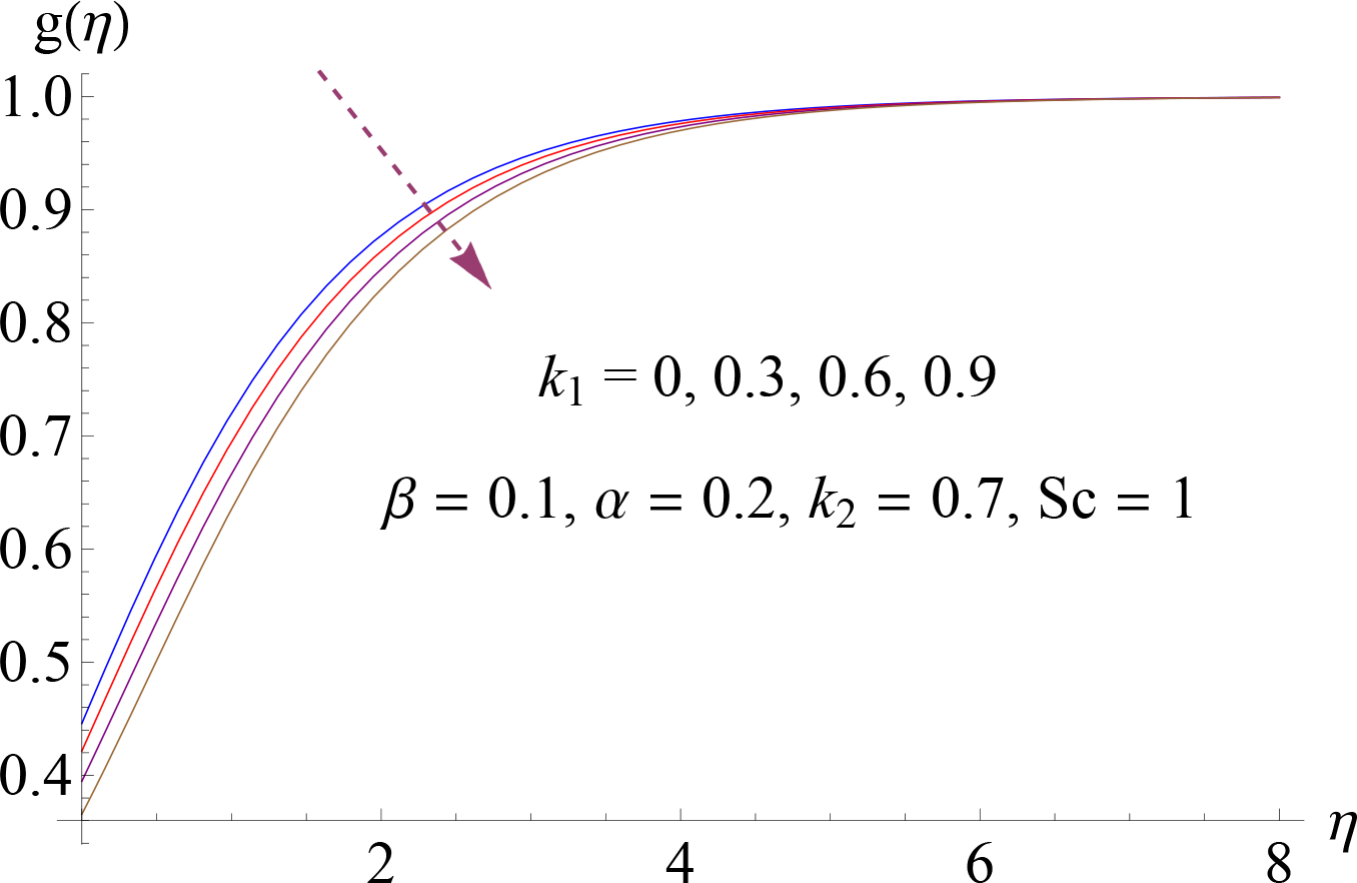
Impact of *k*_1_ on *g*(*η*).

**Fig 9:**
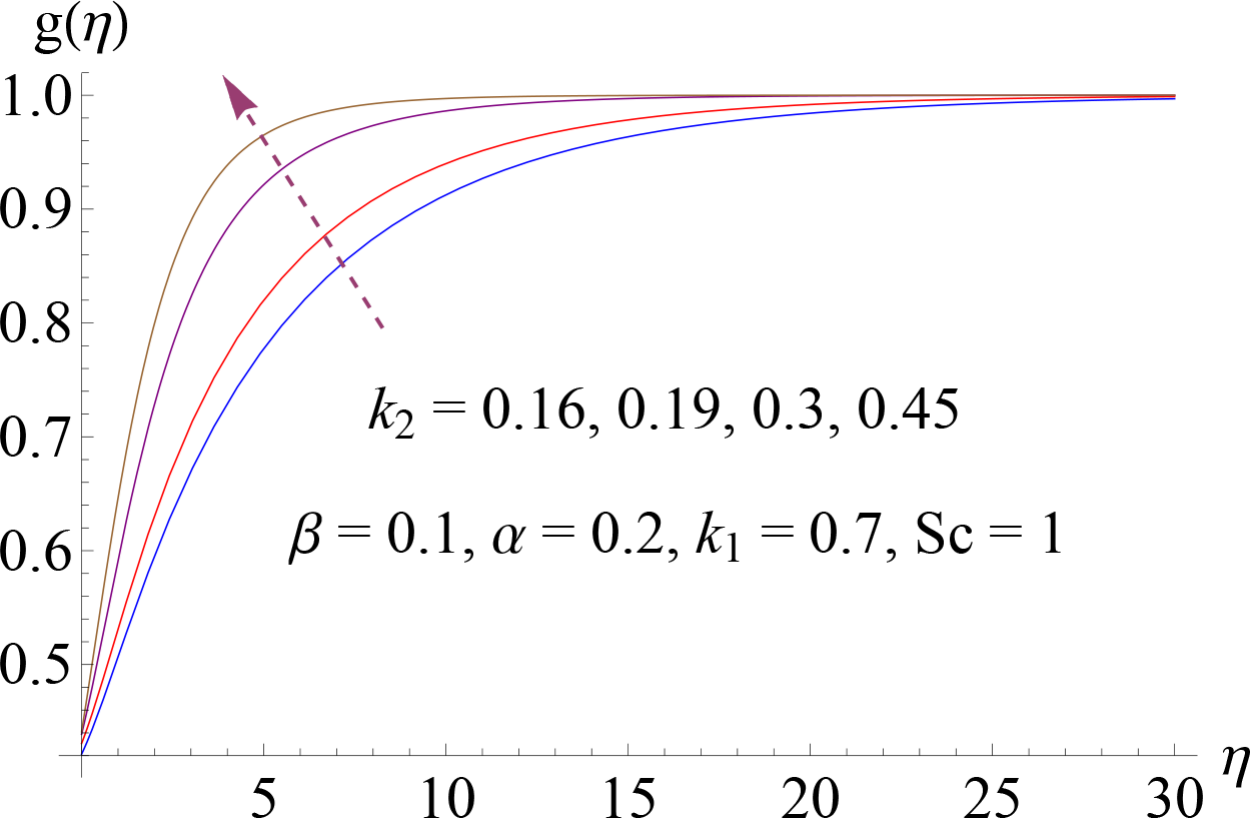
Impact of *k*_2_ on *g*(*η*).

**Fig 10:**
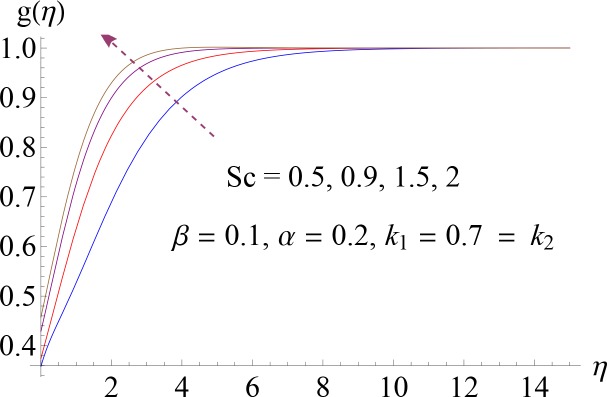
Impact of *Sc* on *g*(*η*).

**Fig 11:**
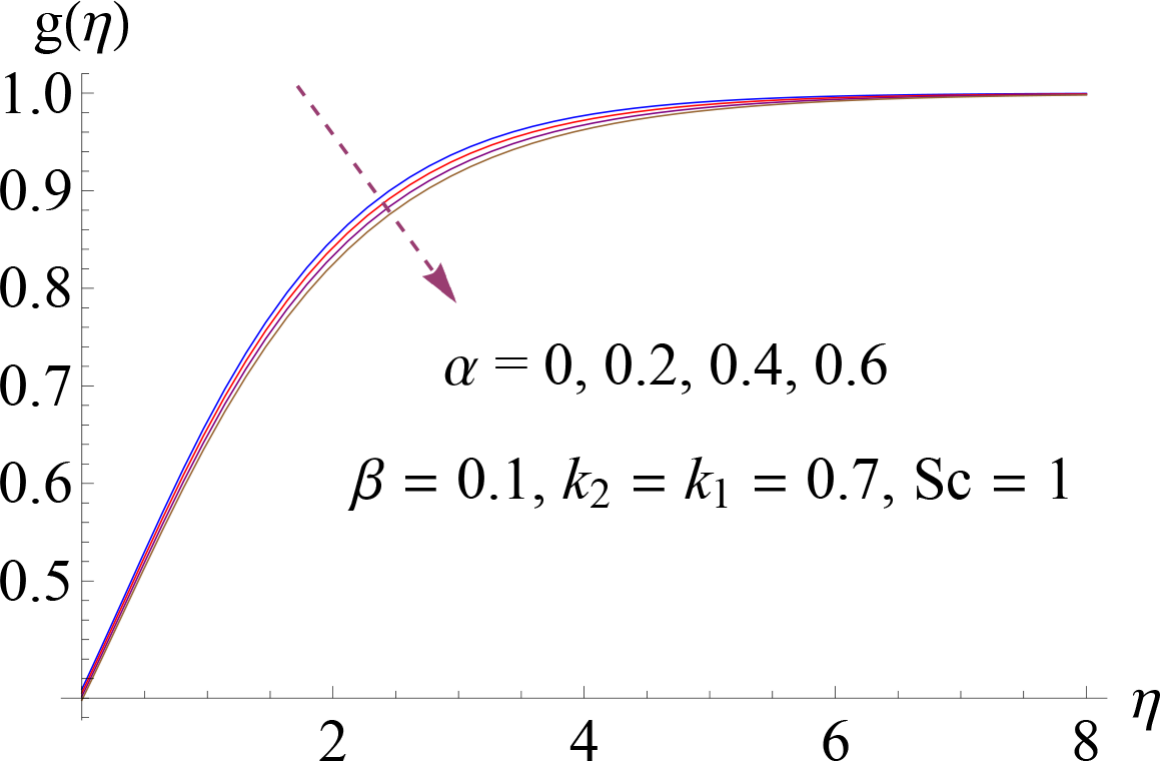
Impact of *α* on *g*(*η*).

**Fig 12:**
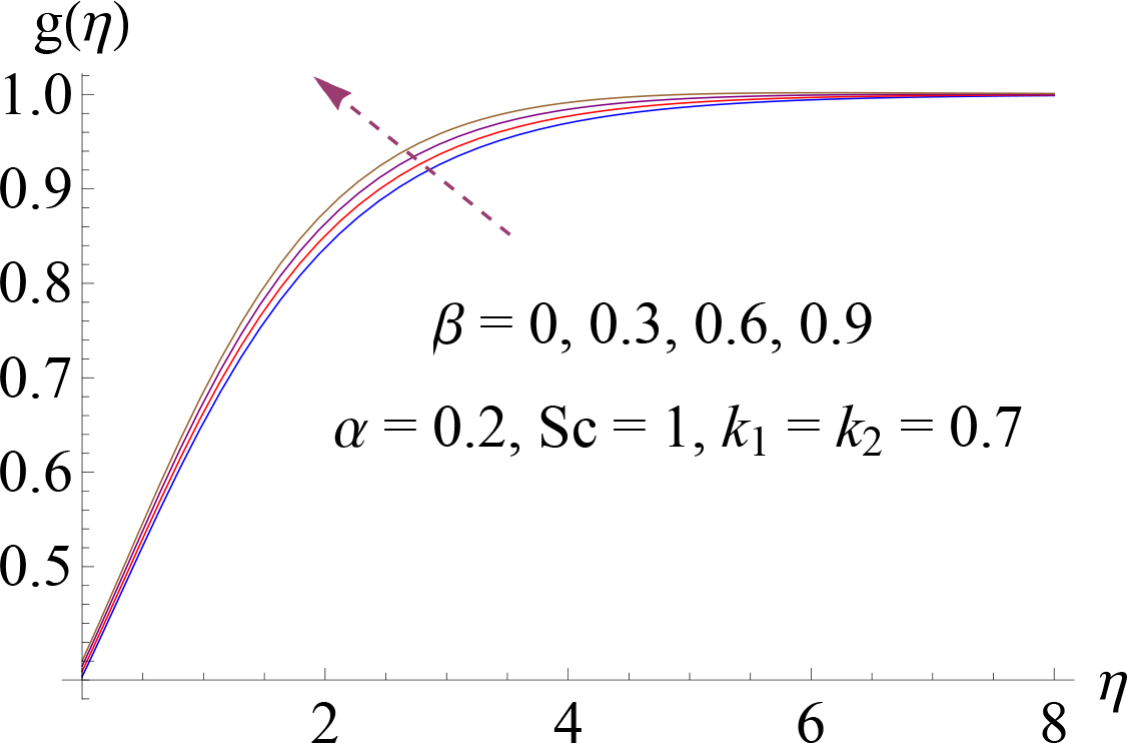
Impact of *β* on *g*(*η*).

**Fig 13:**
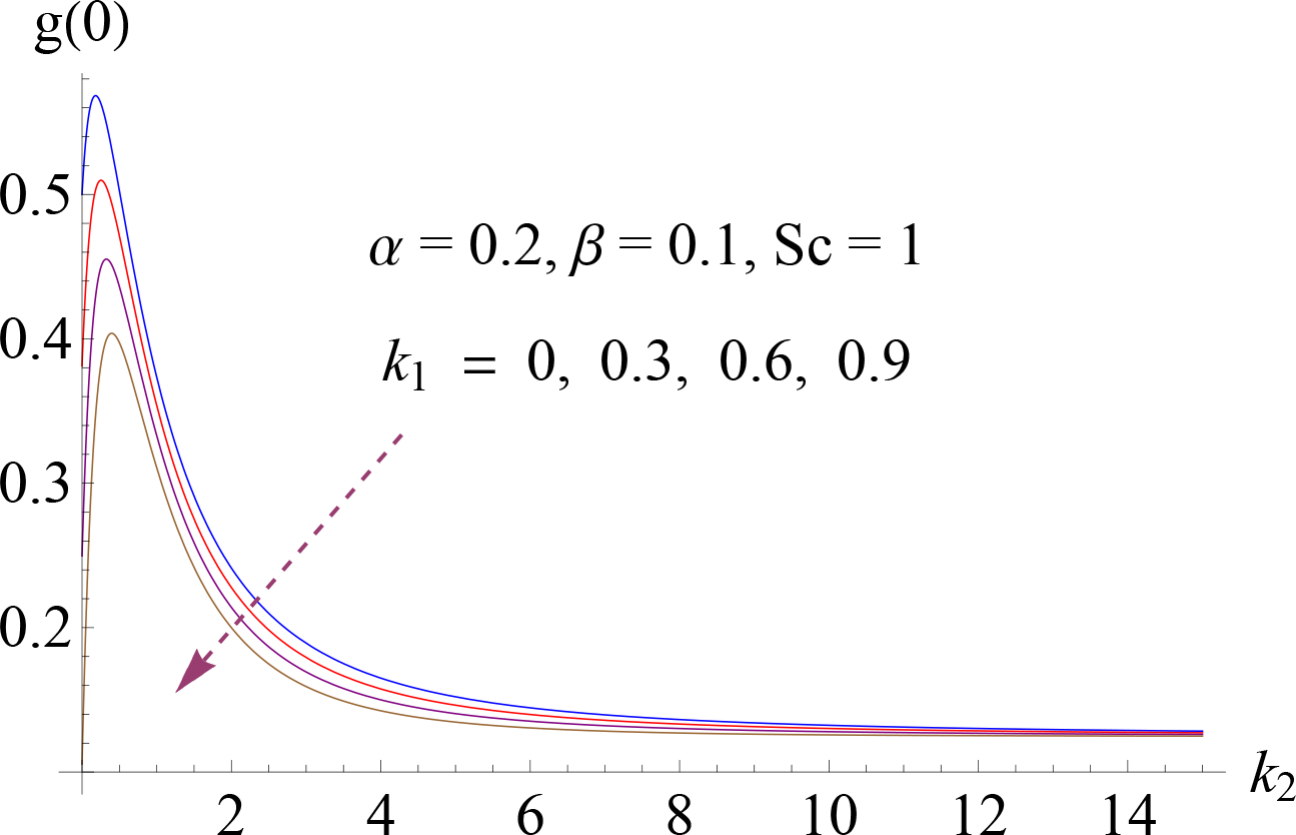
Impact of *k*_1_ on *g*(0).

**Fig 14:**
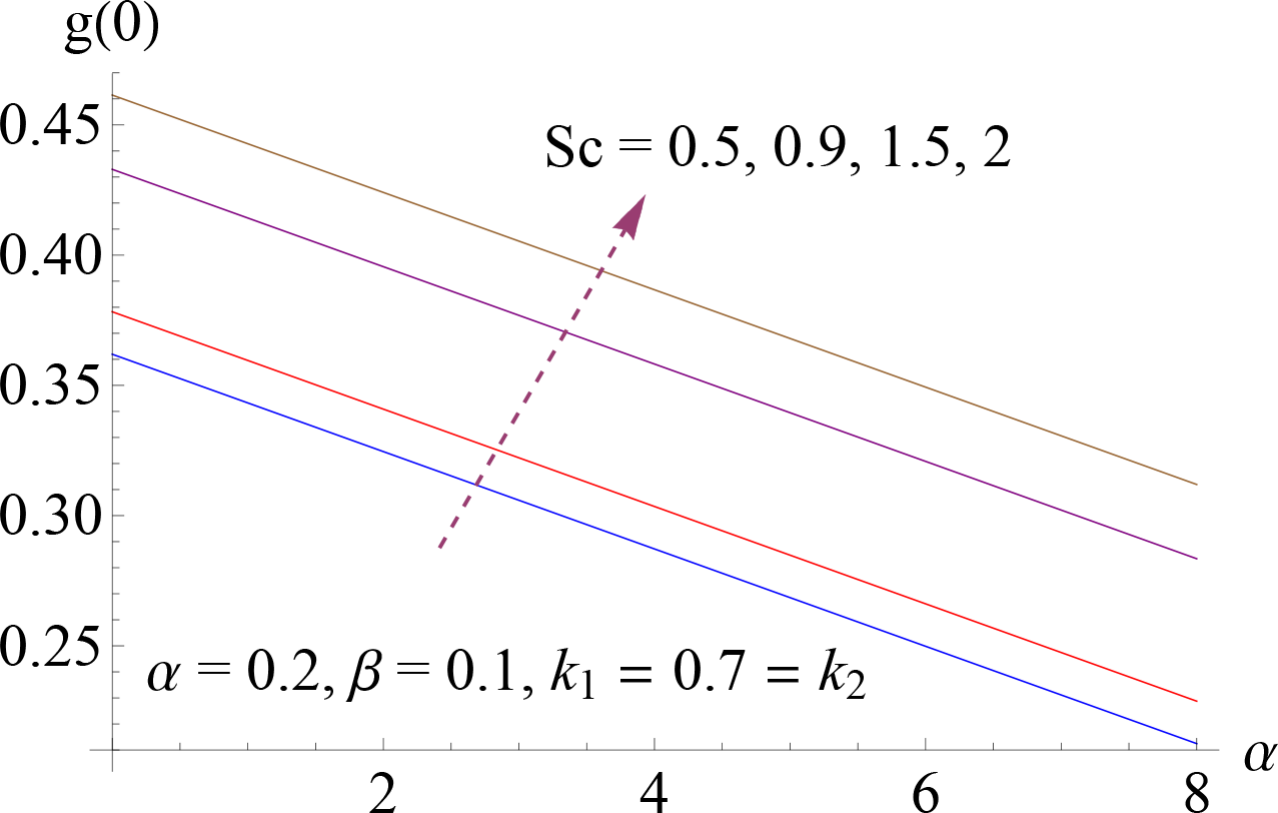
Impact of *Sc* on *g*(0).

**Table 2:** Numerical values of $C_{fx}\;{\text{Re}}_{x}^{0.5}/2$ for various values of the physical parameters.

*β*	*α*	$-C_{fx}\;{\text{Re}}_{x}^{0.5}/2$
0.1	0.2	0.95743
	0.3	0.91987
	0.4	0.88641
	0.5	0.85635
0.2	0.2	1.00000
	0.3	0.96077
	0.4	0.92582
	0.5	0.89442
0.3	0.2	1.0408
	0.3	1.0000
	0.4	0.96362
	0.5	0.93095
0.4	0.2	1.0801
	0.3	1.0378
	0.4	1.0000
	0.5	0.96608

### 5.1. Dimensionless Velocity Profiles

Figs 2 and 3 illustrate the dimensionless velocity profile *f*′(*η*) for several values of ratio of relaxation to retardation times *α* and Deborah number *β*. It is noted that momentum boundary layer decreases via larger *α*. Since *α* is inversely proportional to the retardation time of the non-Newtonian fluid so by increasing *α* there is reduction in retardation time and consequently the fluid flow reduces (Fig 2). Fig 3 represents the impact of various values of Deborah number *β* on dimensionless velocity *f*′(*η*) when *α* = 0.2. Here larger Deborah number leads to an increase in momentum boundary layer. It is due to the fact that *β* and stretching rate of sheet are proportional to each other (i.e. *β* = *λ*_1_*c*).

### 5.2. Dimensionless Temperature Profiles

Figs 4–7 show the impact of increasing values of Prandtl number Pr, thermal relaxation time *γ*, ratio of relaxation to retardation times *α* and Deborah number *β* on dimensionless temperature *θ*(*η*). Fig 4 shows the influence of various values of Pr on fluid temperature. We observed that temperature is decreasing function of Prandtl number Pr. Thermal diffusivity of the fluid layer reduces for larger Pr. Both temperature and thermal boundary layer decrease. Temperature profile for increasing value of *γ* is shown in Fig 5. Here by increasing thermal relaxation time the temperature and thermal boundary layer thickness decrease. It is due to fact that as we increase the thermal relaxation time parameter, particles of the material require more time to transfer heat to its neighboring particles. In other words we can say that for higher values of thermal relaxation parameter the material shows a non-conducting behavior which is responsible in reduction of temperature distribution. We observed from Fig 6 that temperature increases for higher values of *α*. By increasing *α* we noted that there is increase in relaxation time and decrease in retardation time. Temperature enhances since increase in relaxation time is more than the retardation time. Temperature decreases for larger value of Deborah number (Fig 7). Here *β* = *λ*_1_*c* indicates that retardation time enhances with an increase in Deborah number. Such increase in retardation time corresponds to the decrease in the temperature and thermal boundary layer thickness.

### 5.3. Dimensionless Concentration Profiles

Figs 8–12 show the effect of measure of strength of homogeneous reaction *k*_1_, measure of strength of the heterogeneous reaction *k*_2_, Schmidt number *Sc*, ratio of relaxation to retardation times *α* and Deborah number *β* on dimensionless concentration profile *g*(*η*). Effect of *k*_1_ on concentration profile is shown in Fig 8. It shows that by increasing *k*_1_ there is a decrease in concentration profile (because the reactants are consumed during chemical reaction). Fig 9 shows the impact of *k*_2_ on concentration profile *g*. For increasing value of *k*_2_ the diffusion coefficient reduces and less diffused particles enhance the concentration. Effect of Schmidt number *Sc* on *g*(*η*) is shown in Fig 10. Increasing behavior of concentration profile is noted for larger *Sc*. In fact Schmidt number is the ratio of momentum diffusivity to mass diffusivity. Therefore higher value of Schmidt number correspond to higher momentum diffusivity which in turn enhances the concentration profile. Behavior of ratio of relaxation to retardation times *α* on concentration distribution is analyzed in Fig 11. It is noted that for increasing value of *α* there is decrease in *g*. Fig 12 depicts that concentration profile enhances with an increase in *β*.

### 5.4. Surface Concentration

Fig 13 depicts the influence of strength of homogeneous reaction parameter *k*_1_ on surface concentration *g*(0). One can see from the Fig that by increasing *k*_1_ there is a decrease in *g*(0). Variation of dimensionless wall concentration *g*(0) for different values of Schmidt number *Sc* is shown in Fig 14. It shows that *Sc* is increasing function of *g*(0).

### 5.5. Skin Friction Coefficient

Table 2 depicts the numerical values of $C_{fx}\;{\text{Re}}_{x}^{0.5}/2$ for increasing values of the different involved parameters. It is noted that for increasing *α* the skin friction coefficient decreases while there is increase in skin friction coefficient via larger *β*. The values of shear stress at the surface are compared with previous published results in Table 3. Here it is seen that the obtained solutions agree well with results of Abbasi et al. [7].

**Table 3:** Comparison of $C_{fx}\;{\text{Re}}_{x}^{0.5}/2$ for different values.

*α*	*β*	Abbasi et al. [7]	Present results
0	0.2	1.09545	1.09545
0.5	0.2	0.89443	0.89442
0.7	0.2	0.84017	0.84016
1	0.2	0.77460	0.77460
0.4	0	0.84515	0.84515
0.4	0.3	0.96362	0.96362
0.4	0.6	1.06904	1.06904
0.4	1	1.19523	1.19523

## Conclusions

Cattaneo-Christov heat flux model is used to study the flow of Jeffrey fluid over a stretching sheet. Effects of homogeneous-heterogeneous are taken into account. Key points are as follows:

- Velocity profile is increasing function of Deborah number and decreasing function of ratio of relaxation to retardation times.
- Temperature decreases when Prandtl number and thermal relaxation time are increased.
- Effects of strength of homogeneous and heterogeneous reactions are opposite for concentration distribution.
- Concentration is more via larger Schmidt number.
- Surface drag force reduces when ratio of relaxation to retardation times is increased.
- Present results of surface shear stress agree well with previous published work.
